# IRAG1 Deficient Mice Develop PKG1β Dependent Pulmonary Hypertension

**DOI:** 10.3390/cells9102280

**Published:** 2020-10-13

**Authors:** Siladitta Biswas, Baktybek Kojonazarov, Stefan Hadzic, Michael Majer, Ganimete Bajraktari, Tatyana Novoyatleva, Hossein Ardeschir Ghofrani, Friedrich Grimminger, Werner Seeger, Norbert Weissmann, Jens Schlossmann, Ralph Theo Schermuly

**Affiliations:** 1 Universities of Giessen and Marburg Lung Centre, German Center for Lung Research (DZL), 35392 Giessen, Germany; Siladitta.Biswas@innere.med.uni-giessen.de (S.B.); Baktybek.Kojonazarov@innere.med.uni-giessen.de (B.K.); Stefan.Hadzic@innere.med.uni-giessen.de (S.H.); Tatyana.Novoyatleva@innere.med.uni-giessen.de (T.N.); ardeschir.ghofrani@innere.med.uni-giessen.de (H.A.G.); friedrich.grimminger@innere.med.uni-giessen.de (F.G.); Werner.Seeger@innere.med.uni-giessen.de (W.S.); Norbert.Weissmann@innere.med.uni-giessen.de (N.W.); 2 Institute for Lung Health (ILH), 35392 Giessen, Germany; 3 Department of Pharmacology and Toxicology, Institute of Pharmacy, University of Regensburg, 93040 Regensburg, Germany; Michael.Majer@chemie.uni-regensburg.de (M.M.); Ganimete.Bajraktari@chemie.uni-regensburg.de (G.B.); jens.schlossmann@chemie.uni-regensburg.de (J.S.); 4 Max-Planck-Institute for Heart and Lung Research, 61231 Bad Nauheim, Germany

**Keywords:** IRAG1, PKG1β, Pulmonary Hypertension, PASMC, RV dysfunction

## Abstract

PKGs are serine/threonine kinases. PKG1 has two isoforms—PKG1α and β. Inositol trisphosphate receptor (IP_3_R)-associated cGMP-kinase substrate 1 (IRAG1) is a substrate for PKG1β. IRAG1 is also known to further interact with IP_3_RI, which mediates intracellular Ca^2+^ release. However, the role of IRAG1 in PH is not known. Herein, WT and IRAG1 KO mice were kept under normoxic or hypoxic (10% O_2_) conditions for five weeks. Animals were evaluated for echocardiographic variables and went through right heart catheterization. Animals were further sacrificed to prepare lungs and right ventricular (RV) for immunostaining, western blotting, and pulmonary artery smooth muscle cell (PASMC) isolation. IRAG1 is expressed in PASMCs and downregulated under hypoxic conditions. Genetic deletion of IRAG1 leads to RV hypertrophy, increase in RV systolic pressure, and RV dysfunction in mice. Absence of IRAG1 in lung and RV have direct impacts on PKG1β expression. Attenuated PKG1β expression in IRAG1 KO mice further dysregulates other downstream candidates of PKG1β in RV. IRAG1 KO mice develop PH spontaneously. Our results indicate that PKG1β signaling via IRAG1 is essential for the homeostasis of PASMCs and RV. Disturbing this signaling complex by deleting IRAG1 can lead to RV dysfunction and development of PH in mice.

## 1. Introduction

Pulmonary hypertension (PH) in humans is defined by resting mean pulmonary artery pressure ≥ 25 mm Hg, pulmonary arterial wedge pressure ≤ 15 mm Hg, and pulmonary vascular resistance > 240 dyn × s × cm^−5^ [1,2]. This disease affects about 1% of the global population. This progressive and multifactorial disease results in overload of the right ventricle and eventually right heart failure. The pathogenesis includes vascular wall remodeling, inflammation, and thrombosis [2]. Pulmonary artery smooth muscle cell (PASMC) proliferation plays a major role in vascular wall remodeling [3]. Like many other diseases, intracellular free Ca^2+^ is known to play a role in the pathogenesis of PH. Intracellular free Ca^2+^ is not only necessary for PASMC contraction but also plays an important role in mediating proliferation [4]. PASMCs isolated from mice exposed to chronic hypoxia also showed elevated level of intracellular Ca^2+^ [5].

Studies conducted previously suggested that Nitric Oxide (NO) is involved in etiology in PH. NO is produced by NO synthases (NOS) in vascular endothelial cells and diffused into smooth muscle cells [6]. Once diffused, NO leads to activity of its only known receptor to date, soluble Guanylyl Cyclase (sGC). sGC produces cyclin Guanosine Monophosphate (cGMP) from guanosine triphosphate (GTP) [7]. Production of cGMP activates cGMP-dependent kinases (PKG). PKGs are serine/threonine kinases, which encodes for PKG1 and PKG2 genes. PKG1 has two isoforms—PKG1α and β. Although both α and β isoforms are identical in structure except for their N-terminus, PKG1α is the dominant isoform in pulmonary vascular smooth muscle cells [8,9]. PKG1^−/−^ mice had increased mortality rate and developed cardiovascular and hematopoietic disorders [10,11]. NO/cGMP/PKG signaling pathway is of importance as it is involved in smooth muscle relaxation and cell division [12,13]. Activation of PKG1 have been previously shown to decrease Inositol 1,4,5-triphosphate (IP_3_) mediated release of intracellular Ca^2+^, further involved in anti-proliferative and pro-apoptotic mechanisms. IP_3_ functions via IP_3_ receptor (IP_3_RI) to control intracellular Ca^2+^. Recently, Inositol trisphosphate receptor-associated cGMP-kinase substrate 1 (IRAG1, also known as Mrvi1, Ris1, IRAG) has been identified as a substrate for PKG1β, which is also known to interact with IP_3_RI. These proteins were co-immunoprecipitated and thus confirmed the formation of a macrocomplex [14]. IRAG1 is a 125 kDa protein, which has been shown to express in predominantly in smooth muscle cells [15]. When isolated aortic rings from wild type (WT) and IRAG1^∆12/∆12^ mice (exon 12 were flanked in the mice) were stimulated with phenylephrine (PE), they both show similar contraction but relaxation upon adding 8-Br-cGMP was reduced in IRAG1 mutant mice [16]. Therefore, it is clear that IRAG1 has its role in smooth muscle cell relaxation and intracellular Ca^2+^ regulation. Altered relaxation and Ca^2+^ modulation was also shown to be involved in PH. However, the role of IRAG1 and PKG1β/IRAG1/IP_3_RI macrocomplex has never been studied in the context of PH. cGMP elevating agents are known to be beneficial in pulmonary arterial hypertension although different cGMP signaling pathways are not fully understood [17,18]. Therefore, in this study, we aim to characterize the PKG1β/IRAG1/IP_3_RI macrocomplex under hypoxic condition and investigate the role of IRAG1 in PH by a previously described knock out mice model of IRAG1 [19].

This knock out mice model of IRAG1 was generated by deleting exon 3 to make sure that PKG1β and IRAG1 interaction is abolished. These knock out mice had reduced lifetime. Desch et al. further confirmed that there is no reduction of cGMP production in the smooth muscle of these mice [19]. In this article, we show that IRAG1 is downregulated in hypoxic mice lung and in mice PASMCs kept in hypoxia (1% O_2_). Echocardiography, right heart catheterization, western blotting, and immunostaining revealed that IRAG1-deficient mice develop PKG1β dependent pulmonary hypertension under normoxic conditions.

## 2. Materials and Methods

### 2.1. Genetic Mouse Models

IRAG1^+/+^ (further denoted as WT) and IRAG1^−/−^ (further denoted as IRAG1 KO) mice from 129/Sv background were used in this article. Preparation of these animals were previously described elsewhere [19]. All experiments were performed according to the national and international guidelines and with approval from Federal Authorities for Animal Research of the Regierungspräsidium Giessen (Hessen, Germany), proposal number GI 20/10 Nr. 40/2011.

### 2.2. Hypoxia-Induced Pulmonary Hypertension in Mice

Mice were age matched (8 weeks old) and distributed randomly. These mice were kept in either normoxic or hypoxic condition (10% O_2_) for 5 weeks. We did not observe any mortality in this experimental cohort. Sample size was assessed before conducting in vivo experiments. An expected change under hypoxia in right ventricular systolic pressure (RVSP) was the basis for the sample size calculation. From our previous experiences with WT mice either in normoxia or in hypoxia, we used the following sample size calculation:
Difference in means between the groups-10.8Standard deviation-5.5Number of groups-2Power-90%Alpha-0.05
Based on this, we calculated that the minimum number of mice required per group would be 5.

### 2.3. Echocardiography and Right Heart Catheterization

Anesthesia was induced with 3% isoflurane and maintained with 1.5% isoflurane. Heart rate and body temperature of the mice were monitored over the duration of transthoracic echocardiography. Echocardiography was done using a Vevo 2100 system (VisualSonics, Toronto, ON, Canada) as described [20].

The mice were anesthetized as mentioned before, for catheterization. The right external jugular vein was catheterized with a high fidelity 1.4F micromanometer catheter (Millar Instruments, Houston, TX, USA). Data were collected and analyzed using the PowerLab data acquisition system (MPVS-Ultra Single Segment Foundation System, ADInstruments, Oxford, UK) and LabChart 7.

While acquiring data for Echocardiography, investigators were blinded about the groups and respective animal numbers. Similarly, while executing the right heart catheterization process, investigators were blinded about the groups and not aware of the results derived from Echocardiography.

### 2.4. Tissue Preparation and Histology

After sacrificing the mice, either lung and heart was either formalin fixed (4%) for 24 h and paraffin embedded or lung was snap frozen and ventricles were separated from the whole heart. Right ventricular (RV) was further separated. RV and LV (Left Ventricle) + S (Septum) were weighed and immediately snap frozen. Snap frozen lung and RV were randomly chosen for further western blot experiments.

Remodeling of the lung vasculature was assessed in histology. Lung sections were stained with an anti-α-smooth muscle cell actin antibody (clone 1A4, Merck, Dreieich, Germany) and an anti-human von-Willebrand factor antibody (Dako, Frankfurt am Main, Germany). Stained thin sections were examined by light microscopy, and the color along the perimeter of the vessel was analyzed using a computerized morphometric system (Qwin, Leica, Sunnyvale, CA, USA). Vessels were categorized as non-muscularized (5% SMC actin around the vessel), partially muscularized (5 to 75% SMC actin around the vessel), or fully muscularized (≥75% SMC actin around the vessel), and pulmonary vessels ranging between 20–70 µm were quantified for the muscularization analysis.

H & E (Hematoxylin and Eosin) staining was performed to assess right ventricular hypertrophy and cross section areas of cardiomyocytes and Sirius Red staining was performed to assess fibrosis. These experiments were performed on a different cohort of mice, aged between 13 and 15 weeks, than the cohort that have been used for Echocardiography and right heart catheterization procedure. Standard protocols were followed as mentioned elsewhere [21,22]. Serial sections were performed (4 µm; used for H & E- and Sirius Red staining). For determination of right ventricular (RV) area and cardiomyocyte (CM) cross section area, H & E staining of 5 sections from three different slides per series of each mouse were analyzed. For determination of fibrosis, Sirius Red staining of two or three serial sections per mouse were examined. All the images were taken at 100× magnification and composed with the Mosaix-Tool of Axiovision software (Zeiss, NY, USA). Area measurement of RV, total heart, and CM were performed with AxioVision software (Zeiss). Inner area of RV and total heart area was measured and mean was calculated for each mouse after normalization of the inner area of RV to total heart area. Cross section areas of 25 randomly chosen cardiomyocytes per section were evaluated and the mean for each mouse was determined. Percentage of fibrosis of total heart area was quantified using ImageJ.

### 2.5. Mouse Pulmonary Arteria Smooth Muscle Cell Isolation and Culture

One hundred and twenty-nine/Sv mice (2–3 months old, 20–30 g body weight) were anaesthetized by IP injection containing ursotamin (100 mg/kg body weight, Serumwerk, Saale, Germany), xylazin (20 mg/kg body weight, Ceva Tiergesundheit, Düsseldorf, Germany), and heparin (50 I.E./g body weight, Ratiopharm, Ulm, Germany). Pulmonary arterial smooth muscle cells (PASMCs) were isolated from mouse precapillary pulmonary arterial vessels using iron particles (Sigma-Aldrich, Taufkirchen, Germany) according to a previously reported protocol [23]. Primary calls were cultured for 6–8 days in smooth muscle cell growth medium (Promocell GmbH, Heidelberg, Germany) supplemented with Normocin (Invivogen, Toulouse, France) and 10% FBS (Sigma-Aldrich). After the first passaging, cells were seeded in 6-well plates (100,000 cells/well) and left for 24h to attach followed by 24 h starvation in smooth muscle cell basal medium (Promocell GmbH) without supplements. The cells were further put in a hypoxic (1% O_2_, 5% CO_2_) or normoxic (21% O_2_, 5% CO_2_) condition in growth medium for 72 h. Afterwards the cells were lysed on ice in cell lysis buffer (Cell Signaling Technology, Leiden, Netherlands) containing PMSF. Protein samples were collected and stored at −20 °C until further analysis.

### 2.6. Human Lung Samples and Human Pulmonary Artery Smooth Muscle Cell Culture

Lung samples were collected from IPAH patients and individuals without PAH (mentioned as Donors) according to the protocol approved by the ethics committee at Faculty of Human Medicine of the University Hospital Giessen (Giessen, Germany) according to European IPS registry (AZ 111/08) and DZL Biobank (58/15) and in accordance with national law and with Good Clinical Practice/International Conference on Harmonization Guidelines. Tissues were obtained during lung transplantation. Human tissue donation was approved by the ethics committee of the University Hospital Giessen in agreement to the principles stated in the Declaration of Helsinki. Patients with IPAH had a mean age 35.38 ± 10.85 (years ± SD). Control individuals had a mean age 43.70 ± 10.81 (years ± SD) [24].

Human PASMCs were cultured in smooth muscle cell (SMC) growth medium-2 (SmGM-2) with inclusion of the supplement mix, containing 5% fetal bovine serum, basic fibroblast growth factor (2 ng/mL), epidermal growth factor (0.5 ng/mL), and insulin (5 μg/mL) (PromoCell GmbH).

### 2.7. Western Blotting

Complete RIPA buffer was prepared by adding 1× Protease and Phosphatase inhibitor Cocktail (Thermo Scientific, Dreieich, Germany) with Pierce RIPA buffer (Thermo Scientific). Complete RIPA buffer were directly added to the cell culture plates or snap frozen tissues and then homogenized to isolate protein. The protein was denatured using 4× LDS sample buffer (Thermo Scientific) and 10× Reducing agent (Thermo Scientific). NuPAGE protein gels (Invitrogen, Carlsbad, CA, USA) were used to run the protein and PVDF membrane (Thermo Scientific) was used to transfer the protein from gel. Membranes were incubated with primary antibodies for overnight at 4 °C and with secondary antibodies for 1 h at RT. ECL substrate (GE Healthcare, Chicago, IL, USA) were added directly into the membrane for 2–3 min before capturing image at Amersham Imager 680 (GE Healthcare). Band intensities were also quantified using the software provided with the Imager.

Primary antibodies—Rabbit IRAG1, Rabbit PKG1α, Rabbit PKG1β (kindly provided by Prof. Schlossmann) [15,25], Rabbit IP_3_RI, Rabbit Pan-Actin (Cell Signaling Technology), Rabbit Serca2a, Rabbit pPLN-Thr 17, Mouse PLN (Badrilla, Leeds, UK).

Secondary antibodies—goat anti-rabbit, goat anti-mouse (Thermo Scientific).

### 2.8. Immunocytochemistry

Human PASMCs were plated on glass coverslips and cultured for 72 h. Then, cells were fixed with 3.7% PFA (Merck) and blocked with 5% BSA (Serva, Heidelberg, Germany). Cells were incubated with primary antibodies for overnight at 4 °C in a humid chamber and with secondary antibodies for 45 min at RT. Cells were further stained with DAPI (Sigma) for 20 min and then fixed with coverslip adding the mounting media (Dako). These were allowed to dry overnight at RT and then images were captured using fluorescence microscope (BZ-9000, Keyence, Osaka, Japan).

Primary antibodies used were previously listed in the western blotting section. Secondary antibody used was goat anti-rabbit (Alexa Fluor 488, Thermo Scientific).

### 2.9. Statistical Analysis

All data are expressed as individually acquired values and mean is indicated. Western blot densitometry data were plotted against the Lognormal values that were acquired from densitometry quantification. When comparing two groups, Student’s unpaired t-test was used, lognormality was assessed for these datasets with D’Agostino and Pearson tests, and F-tests were performed to assess the homogeneity of variance. When comparing more than two groups, one-way ANOVA was used, normality was assessed with D’Agostino and Pearson tests, and Brown–Forsythe tests were performed to assess the homogeneity of variance. The significance of the *p*-value was indicated by asterisks (* *p* < 0.05, ** *p* < 0.01, *** *p* < 0.001, **** *p* < 0.0001). All the statistical calculations were performed using GraphPad Prism 6. For the compilation of figures, Microsoft PowerPoint 2016, Adobe Illustrator CC 2018, and Adobe Photoshop CC 2018 were used.

## 3. Results

### 3.1. IRAG1 Is Expressed in PASMCs and Down Regulated upon Hypoxia

**Figure 1:**
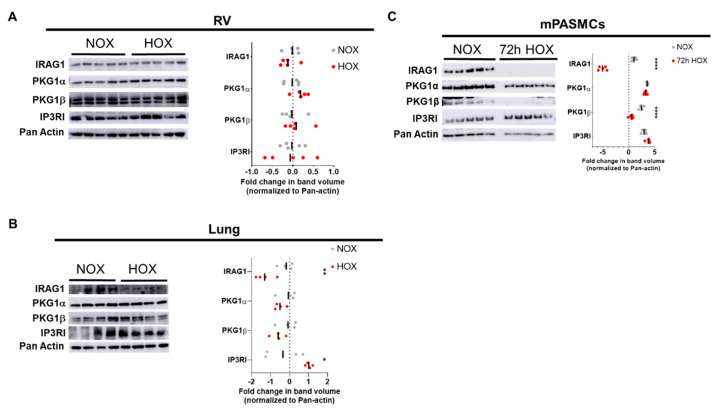
IRAG1 is downregulated in hypoxic mice lungs and isolated mice pulmonary arterial smooth muscle cells under hypoxic condition. (**A**,**B**) Western blots of protein lysates isolated from normoxic and hypoxic mice right ventricles (**A**) and lungs (**B**). Representative blot pictures are on the left and subsequent lognormal values of densitometry quantification are on the right. (**C**) Smooth muscle cells isolated from mice pulmonary arteries were cultured either in normoxia or hypoxia (1% O_2_) for 72 h. Representative western blot pictures of protein lysates from these cells are on the left and subsequent lognormal values of densitometry quantification are on the right. Densitometry was performed in the same blot pictures as presented here and n number corresponds to different animals in the same experiment. Number of animals used: (**A**) Right ventricles from five normoxic and five hypoxic WT mice; (**B**) lungs from four normoxic and four hypoxic WT mice; (**C**) mPASMCs isolated from five wildtype (WT) mice. Primary antibodies used in western blots were IRAG1, PKG1α, PKG1β, and IP_3_RI. Pan-actin was used as housekeeping gene. Fold change was calculated in relation to normoxic controls. All the western blot quantification data were represented as individual point and bar indicates the mean, * *p* < 0.05, ** *p* < 0.01, *** *p* < 0.001, **** *p* < 0.0001, two-tailed unpaired *t*-Test.

Although it has been previously confirmed that IRAG1 is expressed in lung and heart, the effect on IRAG1 expression in these organs have been not been studied [15]. Therefore, western blot was performed with right ventricle and lung homogenates from WT mice exposed either to normoxia or to hypoxia for five weeks (Figure 1A,B) to confirm the effect of hypoxia on the expression of IRAG1 and its immediate upstream (PKG1) and downstream candidates (IP_3_RI). Western blots from mice right ventricles showed no change in expression of IRAG1 and its upstream and downstream candidates between groups (Figure 1A), but western blots from mice lungs revealed significant downregulation of IRAG1 and upregulation IP_3_RI of under hypoxia (Figure 1B). As it was previously known that IRAG1 is expressed in smooth muscle cells [15], mouse PASMCs were isolated and kept under hypoxia (1% O_2_) for 72 h before harvesting proteins. Western blots from these proteins not only confirmed the expression of IRAG1 in PASMCs, but also showed a significant downregulation of IRAG1 under hypoxic condition (Figure 1C). Interestingly, under hypoxic conditions, only PKG1β, but not PKG1α, was significantly downregulated. Additionally, immunostaining of PASMCs isolated from healthy human lungs (denoted as Donors) also confirm perinuclear as well as cytoplasmic expression of IP_3_RI, PKG1β, and PKG1α (Supplementary Figure S1). Due to lack of specificity of the IRAG1 antibody, IRAG1 subcellular expression could not be confirmed in this case. Together, these data confirmed the expression of IRAG1 and both α and β isoforms of PKG1 in mouse PASMCs. Also, hypoxia induced downregulation of IRAG1 can be accompanied by downregulation of PKG1β.

### 3.2. Genetic Deletion of IRAG1 in Mice Causes PH and Pulmonary Vascular Remodeling

**Figure 2:**
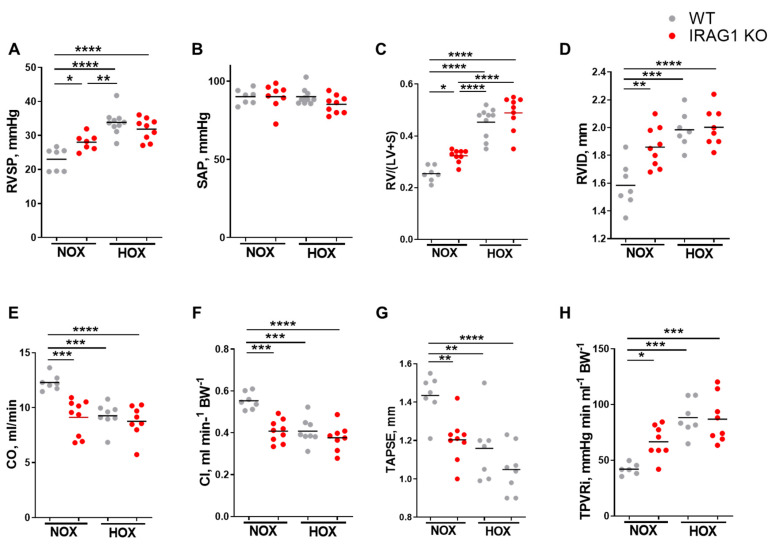
Disruption of IRAG1 gene in mice showed signs of right ventricular dysfunction in normoxic conditions. (**A**–**H**) WT and IRAG1 KO mice were kept under normoxic or hypoxic (10% O_2_) for five weeks and then evaluated for echocardiographic variables and went through right heart catheterization. Variables measured were right ventricular systolic pressure (RVSP, A), systemic arterial pressure (SAP, B), ratio of right ventricular weight to left ventricular wall plus septum weight [{RV/(LV + S)}, C], right ventricular internal diastolic diameter (RVID, D), cardiac output (CO, E), cardiac index (CI, F), Tricuspid annular plane systolic excursion (TAPSE, G), total pulmonary vascular resistance index (TPVRI, H). Number of animals used: (**A**) Normoxia—seven WT and seven IRAG1 KO, hypoxia—10 WT and 9 IRAG1 KO; (**B**) normoxia—seven WT and eight IRAG1 KO, hypoxia—10 WT and 9 IRAG1 KO; (**C**) normoxia—seven WT and nine IRAG1 KO, hypoxia—10 WT and 9 IRAG1 KO; (**D**) normoxia—seven WT and nine IRAG1 KO, hypoxia—seven WT and eight IRAG1 KO; (**E**) normoxia—seven WT and nine IRAG1 KO, hypoxia—eight WT and eight IRAG1 KO; (**F**) normoxia—seven WT and nine IRAG1 KO, hypoxia—eight WT and eight IRAG1 KO; (**G**) normoxia—seven WT and nine IRAG1 KO, hypoxia—seven WT and eight IRAG1 KO; (**H**) normoxia—six WT and eight IRAG1 KO, hypoxia—seven WT and eight IRAG1 KO. All quantified data were represented as individual point and bar indicates the mean, * *p* < 0.05, ** *p* < 0.01, *** *p* < 0.001, **** *p* < 0.0001, comparison of multiple groups were performed by one-way ANOVA, followed by Tukey’s multiple comparisons test between the groups.

**Figure 3:**
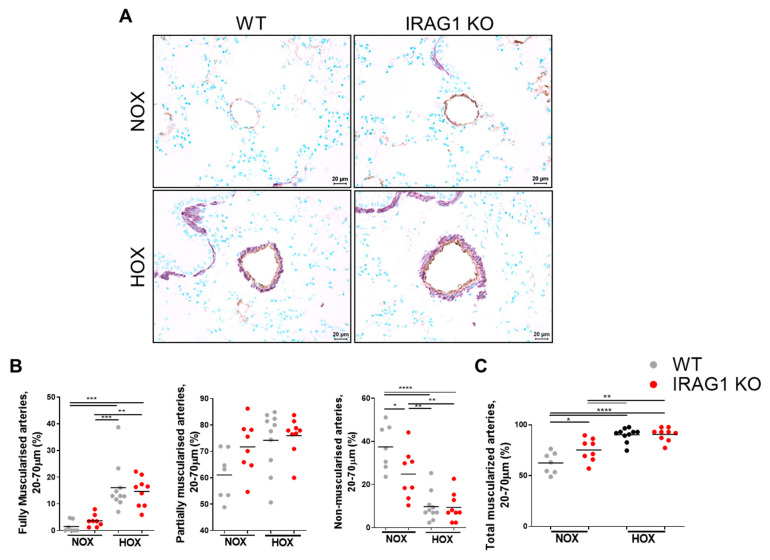
Disruption of IRAG1 gene in mice showed pulmonary artery remodeling in normoxic conditions. (**A**) Formalin fixed paraffin embedded lung sections were immunostained for α-SMA (alpha-smooth muscle actin, violet) and vWF (von-Willebrand factor, brown). Bars indicate 20 μm. (**B**) Quantification of the relative percentage of fully, partially, and non-muscularized arteries were performed. Each group is represented as percentage of the total number of arteries counted. (**C**) Total muscularized arteries were quantified as the percentage of arteries that had any muscularization and represented as percentage of the total number of arteries counted. Number of animals used: (**A**–**C**) Normoxia—seven WT and eight IRAG1 KO, hypoxia—10 WT and 9 IRAG1 KO. All quantified data were represented as individual point and bar indicated the mean, * *p* < 0.05, ** *p* < 0.01, *** *p* < 0.001, **** *p* < 0.0001, comparison of multiple groups were performed by one-way ANOVA, followed by Tukey’s multiple comparisons test between the groups.

**Figure 4:**
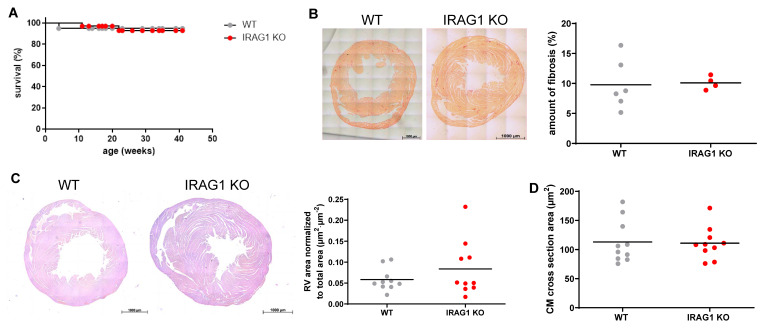
Disruption of IRAG1 gene in mice showed no obvious sign of mortality, increment in right ventricular (RV) fibrosis and area, and cardiomyocytes area. (**A**) Kaplan–Meier analysis was performed to analyze the difference of survival between the two groups. (**B**) Sirius red staining was performed to analyze fibrosis in mice heart and further quantified. Bars indicate 1000 µm. (**C**,**D**) H & E staining was performed with mice heart to analyze RV (**C**) and cardiomyocytes area (**D**) and further quantified. Bars indicate 1000 µm. Number of animals used: (**A**) 20 WT and 35 IRAG1 KO, (**B**) 6 WT and 4 IRAG1 KO, (**C**) 10 WT and 10 IRAG1 KO, (**D**) 10 WT and 10 IRAG1 KO. All quantified data were represented as individual point and bar indicated the mean, two-tailed unpaired *t*-Test.

In line with the previous findings, it was hypothesized that IRAG1 downregulation may play a role in development of PH. Therefore, IRAG1 knock out (KO) mice along with WT mice were kept under normoxic or hypoxic (10% O_2_) conditions and evaluated for echocardiographic variables and went through right heart catheterization (Figure 2A–H). As expected, RV systolic pressure, hypertrophy, dilatation (Figure 2A,C,D), and total pulmonary vascular resistance index (Figure 2H) were significantly increased and cardiac function parameters (Figure 2E–G) were significantly decreased in WT mice under hypoxia. There was no change in the systemic pressure between the groups (Figure 2B), but there was a significant increase of RV systolic pressure in IRAG1 KO mice under normoxia (Figure 2A). Along with this, IRAG1 KO mice also showed RV hypertrophy (Figure 2C) and RV dilatation (Figure 2D) under normoxic condition. To further interest, cardiac index (Figure 2E), cardiac output (Figure 2F), and Tricuspid annular plane systolic excursion (TAPSE, Figure 2G) were all decreased and TPVRI (Figure 2H) were increased in IRAG1 KO mice under normoxia. These data together confirmed the occurrence of PH in IRAG1 KO mice under normoxia. As phenotype of PH is often associated with pulmonary vascular remodeling, immunostaining of mice lungs was performed to identify smooth muscle cells and endothelium (Figure 3A). Upon quantification it can be confirmed that the number of fully muscularized arteries were significantly increased, there were no changes in percentage of partially muscularized arteries, and there was a decrease in percentage of non-muscularized arteries in both groups under hypoxia (Figure 3B). Interestingly, only the percentage of non-muscularized arteries were significantly decreased in IRAG1 KO mice under normoxia (Figure 3B). Therefore, quantification of arteries that had any sign of muscularization i.e., combination of fully and partially muscularized arteries, were performed (Figure 3C). As expected, percentage of muscularized arteries were increased in both groups of mice under hypoxia but IRAG1 KO mice had significantly increased number of muscularized arteries in normoxia (Figure 3C). The IRAG1 KO mice did not have any abnormal mortality rate (Figure 4A), and Sirius red staining confirmed no difference in fibrosis in the heart amongst the groups (Figure 4B). H & E staining in the heart revealed mild but non-significant increase in RV area (Figure 4C); also, cardiomyocyte cross section area remained unchanged between the genotypes (Figure 4D). Overall, these data indicate that IRAG1 KO mice under normoxia have clear signs of RV hypertrophy and dysfunction and mild remodeling of pulmonary arteries.

### 3.3. IRAG1 Deficient Mice Had Endogenous Dysregulation of PKGIβ in Lung and RV

**Figure 5:**
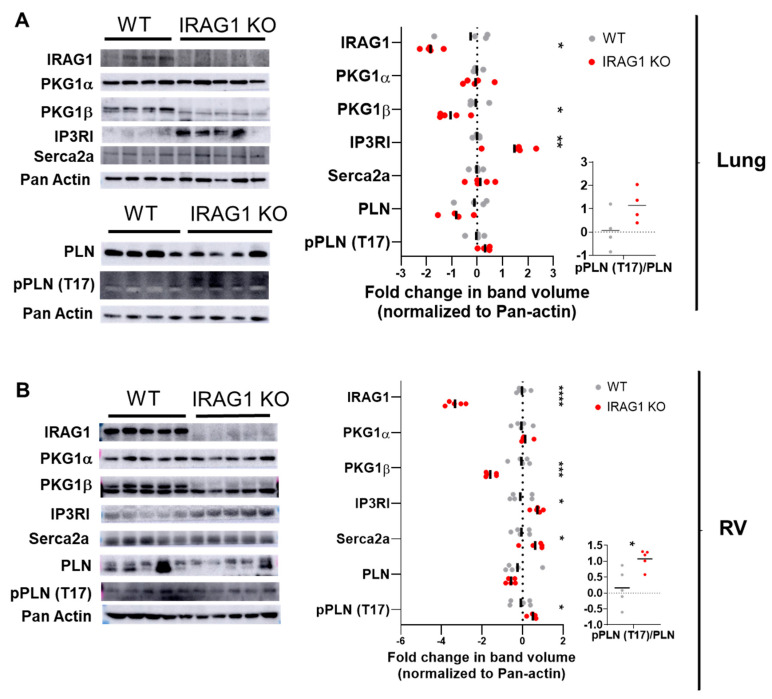
IRAG1-deficient mice had endogenous dysregulation of PKGIβ in lung and RV (**A**,**B**) Western blots of protein lysates isolated from WT and IRAG1 KO mice lungs (**A**) and right ventricles (**B**). Representative blot pictures are on the left and subsequent lognormal values of densitometry quantifications are on the right. Densitometry was performed in the same blot pictures as presented here and n number corresponds to different animals in the same experiment. Number of animals used: (**A**) Lungs from four WT and five IRAG1 KO mice; lungs from four WT and four IRAG1 KO mice for PLN and Ppln (T17); (**B**) right ventricles from five WT and five IRAG1 KO mice. Primary antibodies used in western blots were IRAG1, PKG1α, PKG1β, IP_3_RI, Serca2a, Phospholamban (PLN), and phospho-Thr^17^ Phospholamban [pPLN (T17)]. Pan-actin was used as housekeeping gene. Fold change was calculated in relation to normoxic controls. All the western blot data were represented as individual point and bar indicates the mean, * *p* < 0.05, ** *p* < 0.01, *** *p* < 0.001, ****p < 0.0001, two-tailed unpaired *t*-Test.

To further investigate how IRAG1 is involved at molecular level in development of PH, lung and RV were isolated from WT and IRAG1 KO mice. Homogenates from those lung and RV were analyzed in western blots (Figure 5). As PKG1β, which is upstream of IRAG1, is attenuated under hypoxia and IRAG1 KO mice showed phenotype that is similar of hypoxic mice, it was hypothesized that not only the downstream candidates of IRAG1 but also the downstream targets of PKG1β (other than IRAG1) can also be influenced in IRAG1 KO mice. Lung homogenate western blot showed significant downregulation of IRAG1 and PKG1β, while IP_3_RI was upregulated in IRAG1 KO mice (Figure 5A). Expression of Serca2a and phospholamban (PLN), which are also downstream of PKG1β, were not altered. Similar to lung, in RV, the IRAG1 and PKG1β were also downregulated and IP_3_RI was upregulated in IRAG1 KO mice (Figure 5B). Interestingly, Serca2a but not PLN was upregulated in KO mice. When checked for Threonine-17 phosphorylation of PLN, a significant increase was observed and the ratio of Threonine-17 phosphorylation of PLN to total PLN was higher in RV of IRAG1 KO mice (Figure 5B). To summarize, these western blots elaborate that the absence of IRAG1 in mice influence PKG1β expression in both lung and RV. In RV, the Ca^2+^ regulation pathway that is controlled by PKG1β is also affected in more than one way in IRAG1 KO mice.

### 3.4. IRAG1 and PKG1β Had Stronger Expression in IPAH Patients

**Figure 6:**
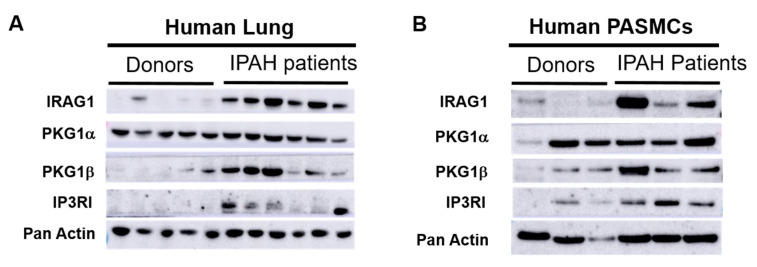
IRAG1 and PKGIβ had stronger expression in lungs and isolated pulmonary artery smooth muscle cells (PASMCs) from end stage IPAH patients. (**A**,**B**) Western blots of protein lysates isolated from human lung homogenate (**A**) and human PASMCs (**B**). (**A**) 5 Donors and 6 IPAH patients lung samples were used. (**B**) 3 Donors and 3 IPAH patients PASMCs were used. Primary antibodies used in western blots were IRAG1, PKG1α, PKG1β, and IP3RI. Pan-actin was used as housekeeping gene.

As Ca^2+^ regulation pathway is often targeted as a therapeutic approach in PH treatment [26,27] investigation with western blots from human lung tissue homogenates (Figure 6A) and pulmonary vascular smooth muscle cell lysates from Donors and end stage IPAH patients (Figure 6B) were therefore performed. These western blots revealed that IRAG1, PKG1β, and IP3RI protein expression were increased while PKG1α expression remained unchanged in patients (Figure 6). Human lung and PASMC data of IRAG1 and PKG1β expression are in contrast to hypoxic mice lung and mPASMCs as described previously, however this contrast makes IRAG1 and PKG1β even more interesting candidates to be further investigated in patient population.

## 4. Discussion

To our knowledge, this is the first time that the cGMP kinase substrate IRAG1 was shown to play a role in RV dysfunction and development of PH under normoxic conditions has been shown. The major novel findings of this article are (1) IRAG1 is expressed in mice PASMCs and downregulated under hypoxic conditions; (2) genetic deletion of IRAG1 leads to RV hypertrophy and dysfunction in mice; (3) absence of IRAG1 in lung and RV have direct impact on PKG1β expression and further dysregulates other downstream candidates of PKG1β.

The importance of the NO-sGC-cGMP pathway in smooth muscle cells in PH has been described previously by Ghofrani et al. [27]. cGMP elevating agents like riociguat have also been proven to be beneficial in phase II and phase III studies with PAH patients although the mechanism is poorly understood [27]. Elevation in cGMP level activates both isozymes of PKG1 and leads to distinct functions. The only known specific substrate of PKG1β is IRAG1 [18]. It has been previously shown that PKG1^-/-^ mice had vascular remodeling in the lung, which further led to PH [28]. Therefore, downregulation of IRAG1 in hypoxic lung kept us interested to explore IRAG1 in smooth muscle cells. Attenuation of both PKG1β and its substrate IRAG1 gave us a clue about the dysregulation of this pathway in hypoxic conditions. Subsequently, we decided to characterize the IRAG1 KO mice in relevance to hypoxic PH model.

In this study, we showed for the first time that IRAG1 KO mice, aged between 8 and 15 weeks, develop mild pulmonary arterial remodeling as the percentage of muscularized arteries were increased in normoxic IRAG1 KO mice. Similarly, RVSP and RV hypertrophy were also mildly increased compared to WT mice. However, the cardiac function parameters and RV dilatation were similar in hypoxic WT mice and normoxic IRAG1 KO mice. Although we did not observe RV fibrosis or cardiomyocyte surface enlargement in IRAG1 KO mice, our echocardiographic examination of the cardiac function indicates impairment of the RV systolic function. The role of fibrosis in the development of RV dysfunction and/or failure is not well defined and might play a dual role as part of an adaptive response at one point and a maladaptive response at another [29]. Prevention of CM stretching is also known to be a part of adaptive response [30]. Also, Fulton index and RVSP in IRAG1 KO mice were not as high as WT mice in hypoxia. Taken together, it can be stated that, although RV function was impaired, RVSP, hypertrophy, and other parameters were not drastically deteriorated. Therefore, it might be that the decreased vasorelaxation promotes vascular remodeling and changes in RV. It is probable that the enhanced pulmonary resistance index leads to changes in the RV. In addition, it might be that direct effects of IRAG1 deletion in the right ventricle influence the IRAG1 KO PH phenotype. Overall, these results confirm the development of PH but at modest level. The most important point here is though the development of spontaneous PH without any additional triggers i.e., hypoxia.

As we found the echocardiographic and hemodynamic parameters were different between WT mice and IRAG1 KO mice under normoxia, it was important to assess the lung and RV for major candidates involved in this pathway. Western blots from lung and RV homogenate of WT and IRAG1 KO mice showed upregulation of IP_3_RI and attenuation of PKG1β. IRAG1 is known to bind with IP_3_RI and reduce Ca^2+^ release from intracellular stores [18]. Hypoxia leads to increment of intracellular Ca^2+^ in PASMCs and knocking down of IP_3_RI can abolish this process [31]. Therefore, it is not surprising that deletion of IRAG1 led to increment of IP_3_RI, which can further stimulate Ca^2+^ release from intracellular stores. It had been shown previously that PKG1β is attenuated in aorta and colon from these same mice that were used in this article [19]. Attenuation of PKG1β in lung and RV of IRAG1 KO mice not only supports these data, but also strengthens the idea that lack of interaction between IP_3_RI and PKG1β in absence of IRAG1 in other organs [19]. Production of cGMP is not affected in the vascular smooth muscle cells of IRAG1 KO mice but acetylcholine mediated relaxation of aorta is reduced [19]. Therefore, PKG1β dependent pathways were expected to be involved. PKG1β not only regulates IRAG1/IP_3_RI mediated Ca^2+^ regulation, but also phosphorylates PLN [32]. Phosphorylation of PLN leads to higher activation of Serca2a and thus increases the import of Ca^2+^ to the sarcoplasmic reticulum [33]. Thr17 phosphorylation site of PLN have been shown to be independent of phosphorylation of Ser16 site in vivo [34]. Total PLN has a reducing trend and PLN-Thr17 phosphorylation is increased in cat aortic banding model of pressure overload hypertrophy and failure [35]. In IRAG1 KO mice lung and RV, we did observe reducing trend of total PLN while PLN-Thr17 phosphorylation is increased in RV. Put together, these results elaborate clear upregulation of IP_3_RI, Serca2a, and PLN-Thr17 phosphorylation in RV of IRAG1 KO mice indicating possible dysregulation of Ca^2+^ regulation.

Overall, our data indicate that IRAG1 KO mice develop RV hypertrophy and functional deterioration in normoxia. Western blots confirm the involvement of other PKG1β dependent pathways involving Ca^2+^ regulation in RV in IRAG1 KO mice. However, as a limitation of this study, we have not been able to specify subcellular localization of IRAG1 in mice or human PASMCs as well as identify which cells in RV expresses IRAG1 and PKG1 isozymes and mechanisms by which they are involved in the Ca^2+^ modulation. It has been shown previously that overexpression of PKG1β in smooth muscle PKG1β rescue/IRAG1 KO mice did not abolish the defect in smooth muscle relaxation (in aorta and colon) [19]. Currently, there are no smooth muscle PKG1β rescue/IRAG1 KO mice available to perform experiments. However, it would be expected from our experiments with these mice in smooth muscle tissues that the effects of PH are not abolished by overexpression of PKG1β in these mice. To our knowledge, this is the first time the IRAG1/PKG1β/IP3RI pathway has been shown in IPAH patients. Therefore, knowledge about this signaling pathway is limited at this point. Most of these patients were in the end stage and have received medications previously that might have an impact on several signaling pathways. Hence, while considering the contrasting results in mice and human tissues and cells, it must be noted that hypoxia is a relatively mild stimulator of PH and does not represent the complete range of idiopathic patients. Higher expression of IRAG1 and PKG1β in IPAH patients can also be speculated as a part of compensatory/recovery mechanism due to previous medications given to these patients. Activation of sGC and several cGMP-elevating agents are known to be beneficial for PH treatment but not known so far to impact IRAG1/PKG1β/IP3RI signaling pathway [36,37,38,39]. Therefore, future research to unravel the role of IRAG1 and PKG1β in lung SMCs and in different cell types of RV may further improve our understanding of cGMP mediated mechanisms, which can also open new avenues for improvement in diagnostic and therapeutic approaches.

## Supplementary Materials

The following are available online at https://www.mdpi.com/2073-4409/9/10/2280/s1, Figure S1: Immunostaining of hPASMCs from Donor confirm perinuclear and cytoplasmic localization of IP3RI, PKG1β, and PKG1α.
