# Supplementary material for: IRAG1 Deficient Mice Develop PKG1β Dependent Pulmonary Hypertension
Source: Cells. 2020 Oct 13;9(10):2280. doi: 10.3390/cells9102280 (PMC7601978; doi:10.3390/cells9102280)
Supplement: Supplementary file 1 [file cells-09-02280-s001.pdf]

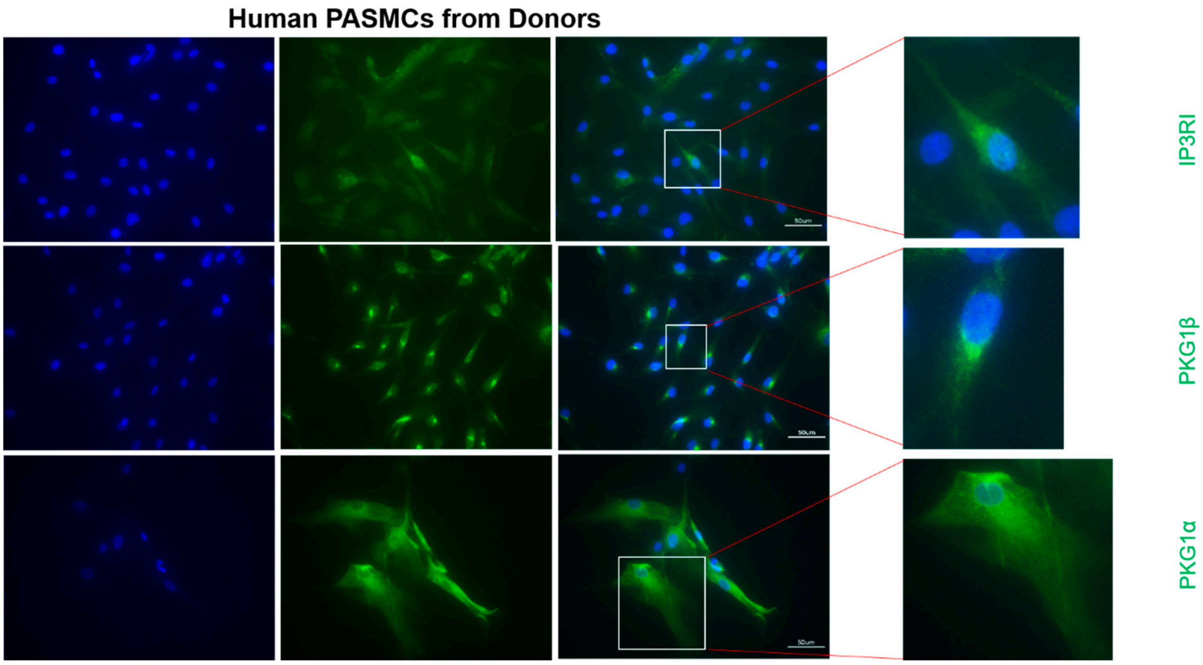

Figure S1. Immunostaining of hPASMCs from Donor confirm perinuclear and cytoplasmic localization of IP3RI, PKG1β, and PKG1α. Primary antibodies used - IP3RI (green, upper panel), PKG1β (green, middle panel), and PKG1α (green, lower panel). Nucleus was stained by DAPI (blue). Scale indicates 50 μm.
